# Interaction of microRNAs with sphingosine kinases, sphingosine-1 phosphate, and sphingosine-1 phosphate receptors in cancer

**DOI:** 10.1007/s12672-021-00430-9

**Published:** 2021-09-20

**Authors:** Guangmeng Xu, Zecheng Yang, Yamin Sun, Hongmei Dong, Jingru Ma

**Affiliations:** 1 Department of Colorectal Surgery, The Second Hospital of Jilin University, Changchun, 130000 China; 2 Department of Gastrointestinal Surgery, The Second Hospital of Jilin University, Changchun, 130000 China; 3 Clinical Laboratory, The Second Hospital of Jilin University, Changchun, 130000 China

**Keywords:** Sphingosine-1-phosphate (S1P), Metastasis, Angiogenesis, MicroRNAs, Cancer

## Abstract

Sphingosine-1-phosphate (S1P), a pleiotropic lipid mediator, participates in various cellular processes during tumorigenesis, including cell proliferation, survival, drug resistance, metastasis, and angiogenesis. S1P is formed by two sphingosine kinases (SphKs), SphK1 and SphK2. The intracellularly produced S1P is delivered to the extracellular space by ATP-binding cassette (ABC) transporters and spinster homolog 2 (SPNS2), where it binds to five transmembrane G protein-coupled receptors to mediate its oncogenic functions (S1PR1-S1PR5). MicroRNAs (miRNAs) are small non-coding RNAs, 21–25 nucleotides in length, that play numerous crucial roles in cancer, such as tumor initiation, progression, apoptosis, metastasis, and angiogenesis via binding to the 3′‐untranslated region (3′‐UTR) of the target mRNA. There is growing evidence that various miRNAs modulate tumorigenesis by regulating the expression of SphKs, and S1P receptors. We have reviewed various roles of miRNAs, SphKs, S1P, and S1P receptors (S1PRs) in malignancies and how notable miRNAs like miR-101, miR-125b, miR-128, and miR-506, miR-1246, miR-21, miR-126, miR499a, miR20a-5p, miR-140-5p, miR-224, miR-137, miR-183-5p, miR-194, miR181b, miR136, and miR-675-3p, modulate S1P signaling. These tumorigenesis modulating miRNAs are involved in different cancers including breast, gastric, hepatocellular carcinoma, prostate, colorectal, cervical, ovarian, and lung cancer via cell proliferation, invasion, angiogenesis, apoptosis, metastasis, immune evasion, chemoresistance, and chemosensitivity. Therefore, understanding the interaction of SphKs, S1P, and S1P receptors with miRNAs in human malignancies will lead to better insights for miRNA-based cancer therapy.

## Introduction

Sphingosine-1-phosphate (S1P) is a bioactive lipid that is produced from the conversion of ceramide to sphingosine and then phosphorylation of sphingosine through sphingosine kinases (SphKs) [1]. To induce cell responses, S1P can either interact with intracellular proteins or be exported from the cell to bind S1P receptors (S1PRs) on cells [2]. The binding of S1P to G protein-coupled receptors (S1PR_1_-S1PR_5_) activates S1PRs and stimulates the associated G proteins to trigger intracellular signals and regulate different processes, such as cell growth, survival, and migration [3]. S1P has a secondary role in the modulation of calcium homeostasis as well. It also enhances the proliferation of cells, survival and inhibits apoptosis [4]. There is also compelling evidence that supports the role of S1P in cancer, including promoting transformation, cell survival, epithelial-mesenchymal transition (EMT), replicative immortality, aerobic glycolysis, and tumor angiogenesis [5–7]. S1P function has also been allied with in cancer inflammatory pathways, cell invasion, and resistance to chemotherapy [8]. S1P-SphK1pathway is involved in head and neck cancer, renal cancer, glioblastoma and leukemias of various types [9]. Mouse models have shown that S1P pathway is also implicated in colon carcinogenesis [10]. Thus, targeting S1P, S1PR and associated proteins in its signaling pathway is a novel therapeutic approach in cancer therapy.

MicroRNAs (miRNAs), with 21–25 nucleotides in length, are a member of non-coding RNAs (ncRNAs) that regulate gene expression via binding to the 3′-untranslated region (3′-UTR) of the target gene, leading to degradation of target mRNA and repression of its translation [11]. Since Lee et al*.* discovered the first miRNA, lin‐4, from *Caenorhabditis elegans* worm [12], more than 2300 mature human miRNAs have been identified [13]. Due to regulate the expression of approximately 90% of human genes, miRNAs are vital factors in controlling cellular processes [14]. There is increasing evidence of crosstalk between miRNAs with SphKs, S1P, and S1PRs that regulates different cellular functions during cancer. This review focuses on the miRNA biogenesis, their involvement in cancer, S1P biosynthesis, and its importance in tumorigenesis and cancer development. Finally, the interaction between miRNAs with SphKs, S1P, and S1PRs is discussed.

## miRNAs biogenesis and their role in cancer

miRNA biogenesis is a complex and multi-step process that starts in the nucleus and undergoes post-transcriptional modifications and then subsequently transported to the cytoplasm to regulate gene expression. In the nucleus, miRNAs are transcribed mostly by RNA polymerase II as mRNAs, while other miRNAs are transcribed by RNA polymerase III, leading to generate the primary miRNA (pri-miRNA) that is capped and polyadenylated at 5´ and 3´, respectively [15, 16]. The hairpin-structured pri-miRNA is recognized and cleaved by the microprocessor composed of an RNase III enzyme, DROSHA, and its cofactor, DGCR8, which produces precursor miRNA (pre-miRNA) with about 60–70 nucleotides in length [17, 18]. Exportin 5 (Exp5) binds to and transports pre-miRNA into the cytoplasm in a RanGTP-dependent manner [19, 20]. In the cytoplasm, another RNase III enzyme, DICER, cleaves pre-miRNA, releasing a miRNA duplex [21]. Then, the miRNA duplex generated through DICER is loaded onto the Argonaute (AGO) protein which selects a strand as mature miRNA (guide strand) and discards the passenger strand (miRNA*) [22]. Finally, the selected strand is loaded into the miRNA-induced silencing complex (miRISC) and directs the complex to target mRNAs in processing bodies (P-bodies) [23].

It has been demonstrated that miRNA expression is dysregulated during human cancers. Indeed, some miRNAs act as oncogenes (OGs), and others act as tumor suppressors (TSs). Interestingly, some miRNAs can act as both OGs and TSs, depending on the specific tissue they express and target genes [24, 25]. Hanahan and Weinberg proposed that cancer consists of six hallmarks, including sustaining proliferative signaling, resisting cell death, evading growth suppressor, activating invasion and metastasis, enabling replicative immortality, and inducing angiogenesis [26]. It is supposed that dysregulated miRNAs affect one or several hallmarks of cancer for tumor initiation and development. Table1 summarizes the role of different miRNAs in various cancers.

**Table 1:** The role of miRNAs in various cancers

miRNA		Target	Cancer	Mechanism of action	Refs.
miR-1246		CCNG2	Breast	↑Proliferation, invasion, drug resistance	[27]
miR-21		PTEN	CRC	↑Tumor growth and invasion	[28]
miR-126		EZH2	Gastric	↑Chemosensitivity	[29]
miR-499a		SOX6	Cervical	↑Chemoresistance	[30]
miR-20a-5p		SMAD4	CRC	↑Invasion and metastasis	[31]
miR-140-5p		VEGF-A	Breast	↓Invasion and angiogenesis	[32]
miR-224		RASSF8	Gastric	↑Tumor growth, invasion, migration	[33]
miR-210		FGFRL1	HCC	↑Angiogenesis	[34]
miR-137		XIAP	Ovarian	↑Apoptosis	[35]
miR-183-5p		PDCD4	Breast	↑Proliferation, ↓Apoptosis	[36]
miR-194		SOCS2	Prostate	↑Metastasis	[37]
miR-181b		Bcl-2	Lung	↓Chemoresistance	[38]
miR-136		Notch3	Ovarian	↓CSCs activity, ↑Chemosensitivity	[39]
miR-675-3p		CXXC4	Gastric	↑Immune escape	[40]

*CCNG2* cyclin-G2, *CRC* colorectal cancer, *PTEN* phosphatase and tensin homolog, *EZH2* enhancer of zeste homolog 2, *SOX6* sex-determining region Y box 6, *SMAD4* drosophila mothers against decapentaplegic protein4, *VEGF-A* vascular endothelial growth factor A, *RASSF8* ras association domain family member 8, *FGFRL1* fibroblast growth factor receptor-like 1, *HCC* hepatocellular carcinoma, *XIAP* X-linked inhibitor of apoptosis, *PDCD4* programmed cell death protein 4, *SOCS2* suppressor of cytokine signaling 2, *CSCs* cancer stem cells, *CXXC4* CXXC finger protein 4

## S1P biosynthesis and its receptors

**Fig. 1:**
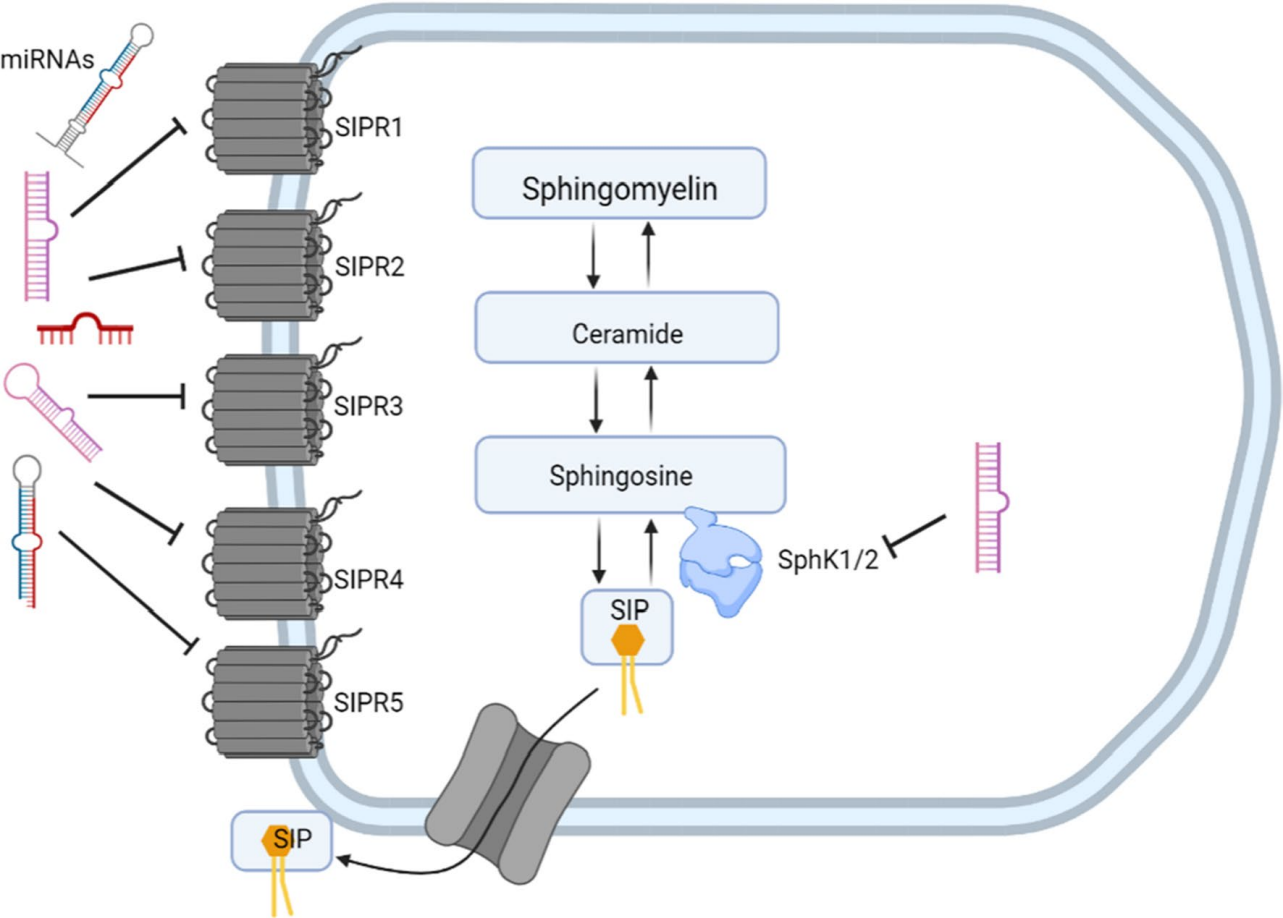
Schematic representation of the biosynthesis process of Sphingosine-1-phosphate (S1P)

To generate S1P, two enzymatic reactions take place inside the cell: first, the ceramide is converted to sphingosine by ceramidase, and then the SphK enzymes convert sphingosine into its phosphorylated form (S1P) using ATP [2]. S1P can inhibit ceramide-mediated apoptosis (including intranucleosomal DNA fragmentation and other structural changes), which occurs in response to the accumulation of ceramide [41]. There are two isoforms of SphK: SphK1 and SphK2. SphK1 is localized close to the cell membrane in the cytosol, where it participates in its substrate's transport [42]. Cytokine-mediated activation of SphK1 leads to the phosphorylation of Sph. This generates intracellular S1P which is then exported from cells by Sphingolipid Transporter 2 (SPNS2) [43]. On the other hand, SphK2 is located in the mitochondria and nucleus. In the mitochondria, S1P can interact with prohibitin 2 to regulate the assembly of complex IV and respiration [44], whereas it regulates the transcription of genes via epigenetic modulations in the nucleus [45, 46]. Thus, S1P generated by SphK1 can export to the outside of the cell to trigger “inside-out” signaling, while S1P generated by SphK1 and SphK2 act intracellularly to regulate cellular functions. “Inside-out” signaling indicates that inside the cell-produced S1P is transported outside the cell to act as autocrine or paracrine via binding to its receptors [47]. Figure 1 shows the biosynthetic process of S1P.

**Fig. 2:**
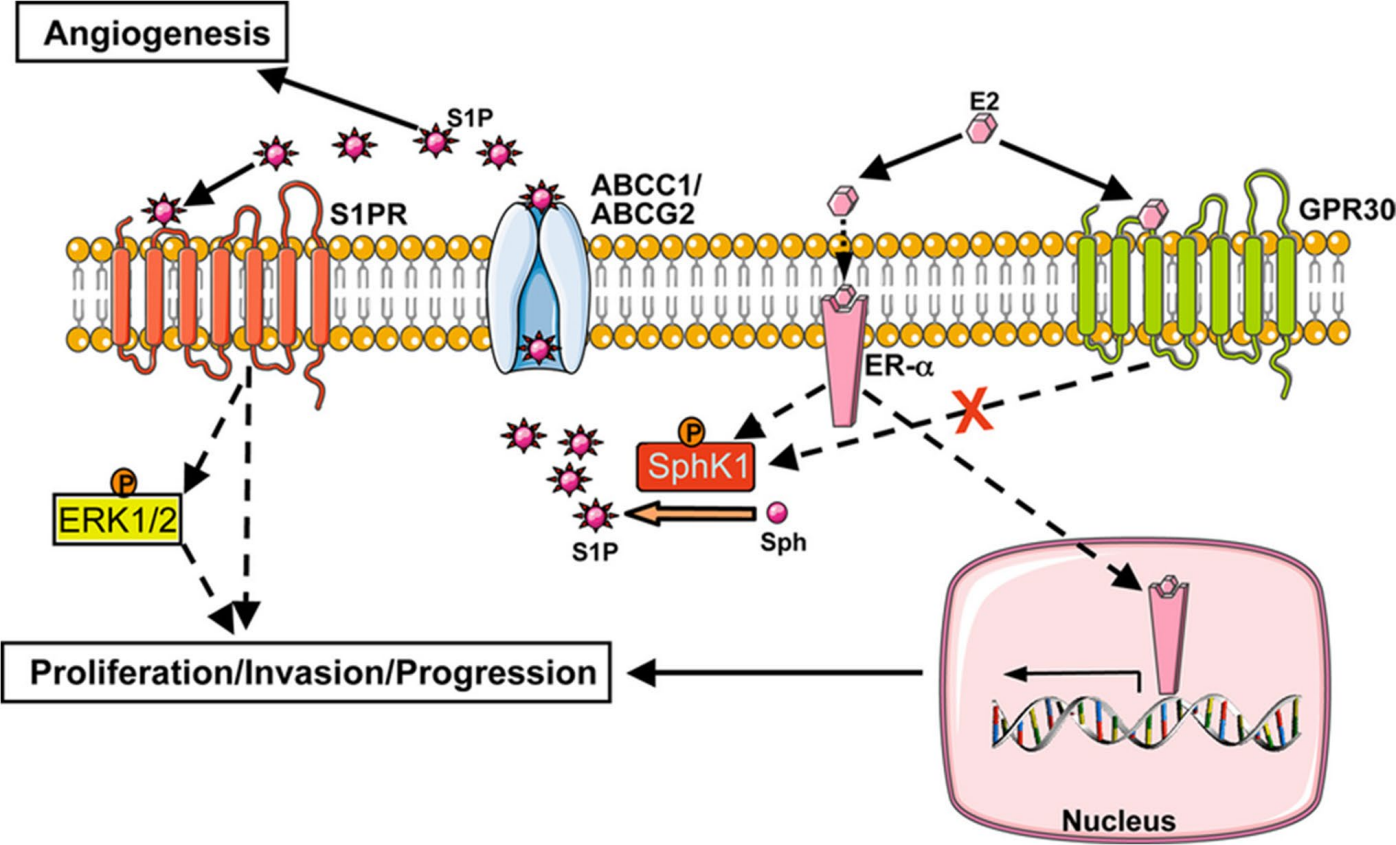
The role of ABC transporters and S1P/dihydro-S1P export from the cell in the nongenomic effects of E2 is depicted in this diagram. S1P (and dihydro-S1P, not shown here) is released via ABC transporters ABCC1 and ABCG2 when E2 binds to ER- but not GPR30. This S1P then binds to and activates S1P receptors, causing ERK1/2 to be activated, resulting in downstream signaling events that are critical for breast cancer proliferation, development, and invasion. (Adopted from [45])

Given to a polar head group, S1P is unable to pass through the plasma membrane freely. It has been demonstrated that ATP-binding cassette (ABC) transporters are involved in S1P export from cancer and non-cancer cells. For example, ABCC1 (MRP1; multidrug-resistant protein 1) transports S1P from mast cells [48], ABCA1 from astrocytes [49], ABCA1 and ABCC1 from endothelial cells [50], and ABCC1 and ABCG2 from breast cancer cells as presented in Fig. 2 [51]. Moreover, spinster homolog 2 (SPNS2), belonging to the non-ATP-dependent organic ion transporters family, also mediates S1P transport [52, 53]. Bradley et al*.* revealed that SPNS2 plays a critical role in S1P transport in lung cancer cells, while its inhibition abolished cancer cell survival and migration [54]. It has been shown that S1P is determined at higher levels in lymph and blood than in tissue [55]. However, two enzymes reduce and control S1P levels: S1P phosphatase and S1P lyase [56]. S1P phosphatase 1 (SGPP1) removes the phosphate group of S1P and converts it to sphingosine which is subsequently converted to ceramide by ceramide synthase [57]. S1P lyase irreversibly cleaves C2–C3 bond in S1P to produce hexadecenal and phosphoethanolamine [58, 59].

High-affinity binding of exported S1P to five transmembrane GPCRs mediates downstream signaling pathways, including pathways mediated by Jun N-terminal kinase (JNK) [60], adenylate cyclase [61], PI3K and Akt [62], and GTPases of the Rho family [63]. The diversity of responses mediated with S1PRs1-5 such as heart development, angiogenesis, cell migration, and immunity, depends on the downstream effectors and receptor expression pattern. For instance, binding to S1PR_1_ or S1PR_3_ can increase the migration of endothelial cells, whereas activation of S1PR_2_ showed an opposite effect [64, 65].

## Roles of S1P and its receptors in cancer and cancer therapy

Since the important role of S1P was demonstrated in inhibiting apoptosis and enhancing proliferation [41, 66], many studies have focused on S1P and its receptor's role in cancer and cancer therapy.

### SphKs and cancer

There is substantial evidence suggesting the crucial role of dysregulated SphKs (specifically SphK1) along with a transformed signaling mechanism, in cancer development. For example, Acharya et al*.* found that SphK1 promoted metastasis of triple-negative breast cancer (TNBC) by upregulating the expression of the *FSCN1* (fascin) gene through NF-κB activation. They showed that the activation of the SphK1/NF-κB/FSCN1 pathway was associated with poor patient survival and distance metastasis, whereas inhibition of SphK1 or NF-κB remarkably inhibited tumor growth and lung metastasis in a TNBC mouse model [67]. SphK1 also leads to the enhanced growth factor receptor (EGFR) transactivation, via estrogen (E2) induction in human breast cancer cells. The mechanism is based on a novel signaling mechanism where E2 triggers activation of SphK1 and as a result, S1P is released. This results in the EGFR transactivation, revealing the role of SphK1 in the signal pairing between S1P, E2, and EGF triggered events [68, 69]. It has been reported that 31% of patients with colorectal cancer (CRC) exhibited higher SphK1 expression and it was correlated with higher histological grade, vascular invasion, lymphatic invasion, distance metastasis, and lower overall survival times [70]. Lee et al*.* revealed that lipopolysaccharide (LPS), the main component in the membrane of Gram-negative bacteria, could activate SphK1 by inducing the S225 phosphorylation of SphK1 and its translocation to the plasma membrane, resulting in S1P production and S1PR_4_ receptor activation which enhances invasion and metastasis of prostate cancer cells [71]. In another study, the comparison of SphK1 expression levels in tumor tissues and adjacent normal tissue samples revealed that patients with hepatocellular carcinoma (HCC) had significantly upregulated SphK1 levels, and higher SphK1 expression was correlated with shorter overall survival times. Furthermore, higher expression of SphK1 in HCC cell lines mediates resistance to oxaliplatin via modulating Akt/GSK3β pathway, while SphK1 knockdown increases chemosensitivity of HCC cell lines [72]. Over expression of SphK1 is also involved in resistance to daunorubicin and cisplatin in leukemia and colon cancer cells [73, 74].

**Fig. 3:**
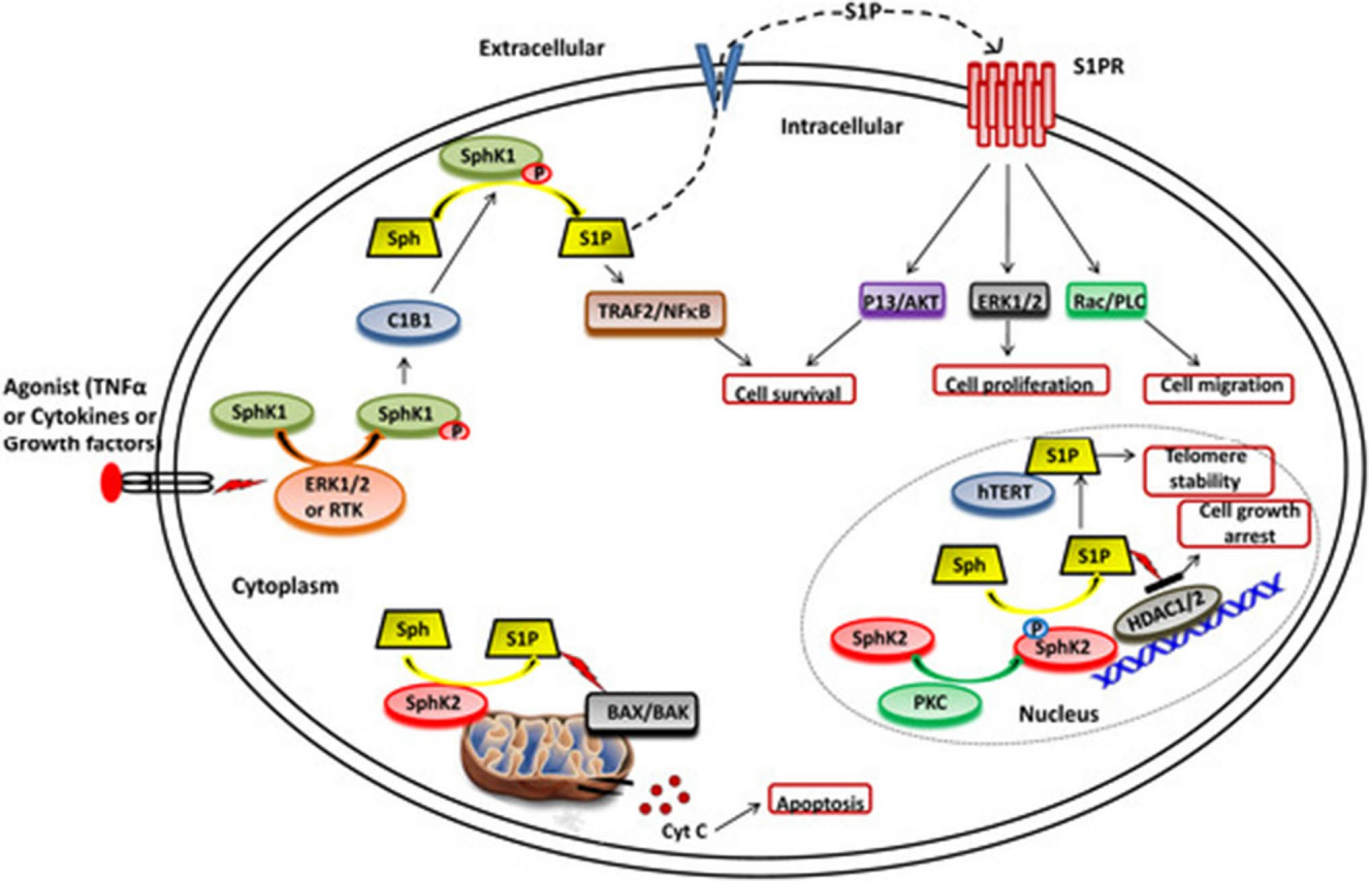
SphKs and S1P have different functions in cells. SphK1 is translocated from the cytoplasm to the plasma membrane and interacts with calcium-myristoyl switch protein 1 after ERK1/2 phosphorylation/activation in the presence of different agonists (such as TNF, cytokines, and other growth hormones) (C1B1). This allows for the phosphorylation of sphingosine to produce S1P, which can then be released or interact with intracellular targets (such as TRAF2) to perform its functions. S1P interacts to the S1P receptor (S1PR) located in the plasma membrane after being secreted out of the cell, activating numerous downstream signalling pathways that affect cell survival, proliferation, and migration. SphK2 facilitates the phosphorylation of sphingosine in the nucleus to produce S1P, which inhibits histone deacetylases (HDAC1/2) and controls gene expression. In human and mouse fibroblasts, S1P binds to human telomerase reverse transcriptase (hTERT) in the nuclear periphery, inhibiting its interaction with makorin ring finger protein 1 (MKRN1) and promoting telomerase stability. SphK2 also causes S1P to be produced in the mitochondria. (https://encyclopedia.pub/10045)

It has been shown that SphK1 could suppress apoptosis of cancer cells. For instance, Taha et al*.* found that SphK1 knockdown led to cell cycle arrest and induction of apoptosis as shown in Fig. 3, characterized by caspase activation, cytochrome c (Cyt c) release, and oligomerization of Bax in the mitochondrial membrane of breast cancer cells [75]. Song et al*.* found that the expression of SphK1 was significantly increased in non-small cell lung cancer (NSCLC) patients which was correlated with poor survival of patients. They also showed that over expression of SphK1 markedly inhibited docetaxel or doxorubicin-induced apoptosis via inducing anti-apoptotic proteins such as Bcl-xl, TRAF1, c-IAP1, and c-IAP2 as indicated in Fig. 3, whereas SphK1 inhibition or silencing enhanced NSCLC cells sensitivity to chemotherapy-induced apoptosis [76]. It has been demonstrated that SphK1 contributes to apoptosis resistance via regulating the Akt/FOXO3a/Bim pathway in glioma cells and gastric cancer cells, leading to the downregulation of Bim as a pro-apoptotic protein [77, 78].

Due to its role in tumor growth and progression, some SphK inhibitors have been developed to combat cancer. For example, MP-A08 reduces S1P levels via selectively targeting the ATP-binding site of SphK1 and SphK2, leading to inhibition of cancer cell proliferation and apoptosis induction [79]. A dual SphK1/SphK2 inhibitor, called SphKI-II, enhances the sensitivity of HCC cells to 5-Fluorouracil (5-FU) and reduces cell proliferation and migration as well as inducing apoptosis [80]. It has been reported as toxic for bladder carcinoma and breast cancer cells [81]. SK1-I, an inhibitor of SK1, induces apoptosis of leukemia cells [82]. LCL146 and LCL351 are SphK activity inhibitors and are harmful to cancer cells [83]. FTY720 vinyl phosphonate inhibits SK1 in breast and prostate cancer cells [84]. Dai et al*.* reported that ABC294640, an SphK2 inhibitor, could suppress Kaposi sarcoma-associated herpesvirus (KSHV)-induced tumor growth by inducing autophagic death instead of apoptosis [85]. A phase I clinical trial indicated that ABC294640 was well tolerated in patients with solid tumors [86]. Patwardhan et al*.* reported reduced S1P levels in lymphoma cultured cells by the action of SLP7111228, an SphK1-selective inhibitor, and SLP120701, an SphK2-selective inhibitor [87].

### S1P, its receptors, and cancer

The five S1PRs show presence in several of the human organs/systems. Initially, Wang et al. [88] reported the presence of S1PR_3_ in nucleus only, while various studies reported that rest of them exist in both the nucleus and cytoplasm. However, Park et al. [89] has reported S1PR3 presence in apoptotic exosome-like vesicles, lumen and membrane as well. Presence of S1PRs on cellular membranes is limited and the expression pattern of all these varies from cell to cell and tissue to tissue, in benign and invasive cells [88]. S1PRs play various roles in cancer [90]. S1PR_1_ enhances the migratory ability of the proliferating cells in a kidney-derived tumor cell line wiT49 [91]. Apart from this, it also enhances the invasion and migration of cancerous cells in glioblastoma [92] and oncogenic fibrosarcoma cell line [93]. S1PR_2_ plays a dual role, with pro-oncogenic and inhibitory properties. It suppresses migration in glioblastoma [94], invasion in melanoma cells [95], and proliferation in Wilms tumor cells [96] while it enhances proliferation in liver cancer cells [97], invasion in glioblastoma cell lines [98], and migration in oral squamous cell carcinoma [99]. Ponnusamy et al*.* demonstrated that the activated S1PR_2_ facilitates lung colonization/metastasis through S1P/S1PR_2_/Brms1 (breast carcinoma metastasis suppressor 1) pathway [100]. On the other hand, it has been shown that S1P reduces hepatocyte growth factor (HGF)-induced migration of HCC via S1PR_2_, but not through other S1PRs [101]. Furthermore, Du et al*.* identified S1PR_2_ as a negative regulator of tumor angiogenesis and growth [102].

SIPR_3_ enhances migration and invasion in gastric [6], breast [103], and nasopharyngeal cancer cell lines [104]. S1P enhances invasion and migration of breast cancer cells both in vitro and in vivo by inducing the expression of matrix metalloproteinase-9 (MMP-9) in the S1P/S1PR_3_/G_αq_ axis [103]. MMP9 facilitates the migration and invasion of cancer cells via cleaving extracellular matrix (ECM) proteins [105]. Recently, Sukocheva et al*.* have reported SphK1 and S1P3 nuclear translocation due to the action of S1P and estrogen results in the cell proliferation in MCF-breast cancer cell lines and vice versa on inhibition [106]. The S1P/S1PR_3_ interaction also mediates metastasis of TNBC via promoting the Notch signaling pathway [107]. It has also been found that S1PR_4_ is associated with poor prognosis and reduces the survival time in estrogen receptor-negative breast cancer cells. S1PR_5_ is also implicated in both suppressing tumors as in squamous cell carcinoma and promoting autophagy in prostate cancer [108].

As mentioned above, there is evidence that S1PRs are involved in tumor angiogenesis. Angiogenesis, sprouting new vessels from the preexisting ones, provides oxygen and nutrients for distant tumor cells to guarantee their growth [109, 110]. Sphingomab®, a monoclonal antibody against S1P, has been developed to block S1P interaction with SIPRs. The anti-S1P monoclonal antibody could reduce tumor proliferation, growth, invasion, and angiogenesis [111]. Sphingomab antibody neutralization of the S1P has been reported to reduce the tumor growth in the murine model. This was also effective in treating the mice resistant to sunitinib treatment [112], probably because it binds with higher affinity than receptors of S1P. This prevents interaction with S1PRs. Old antibodies are no longer available and new ones are being developed [113]. It has been shown that FTY720, an antagonist of S1PRs, could inhibit tumor growth, metastasis, vascularization, and angiogenesis by internalizing S1PRs, inhibiting their recycling to the cell surface, and desensitizing their function [114]. Chae et al*.* developed RNA interference against the S1PR_1_ receptor and found that the S1PR_1_ is required for endothelial cell (EC) migration and tumor angiogenesis, whereas S1PR_1_ silencing suppresses angiogenesis and tumor growth in vivo [115]. Mechanistically, S1P activates S1PR_1_ amplifies VEGFR2‐dependent c‐Abl1 and Rac activation and EC migration to support tumor growth [116]. Dai et al*.* found that S1PR_1_ and S1PR_3_, but not S1PR_2_, mediate the angiogenic effect of S1P. The inhibition of S1PR_1_ and S1PR_3_, but not S1PR_2_, could attenuate tumor angiogenesis and inhibit the expression of angiogenic factors. They also showed that the expression level of SphK1, but not SphK2, was positively correlated with the microvascular density of ovarian cancer tissue [117]. In contrast to the mentioned studies, Gaengel et al*.* indicated that S1PR_1_ signaling restricts angiogenesis, whereas loss of S1PR_1_ increases EC sprouting and vessel branch formation [118].

S1P and S1PRs also mediate the immune escape of cancer cells. For example, Liu et al*.* showed that S1PR_1_ could promote tumor-associated regulatory T cell (Treg) expansion in bladder cancer patients, leading to poor overall survival. Mechanistically, the S1PR_1_ receptor activates TGF-β signaling pathway results in TGF-β and IL-10 secretion from tumor cells. The secreted TGF-β and IL-10 could promote Treg migration into the tumor microenvironment [119]. Chakraborty et al*.* demonstrated that S1P could promote T cells' differentiation into Tregs in a receptor-independent pathway and intracellular peroxisome proliferator-activated receptor gamma (PPARγ)-dependent manner. They showed that SphK1-deficient T cells reduce differentiation to Tregs and inhibition of SphK1 improves the anti-tumor activity of T cells [120].

## Interaction of miRNAs with SphKs, S1P, and its receptors in cancer

There is accumulating evidence that miRNAs mediate tumor development, progression, and treatment via targeting SphKs, S1P, and S1P receptors (Fig. 1). Interaction with various miRNAs has been summed up below.

### miR-148a

MiR-148a is located on chromosome 7p15.2 (68 nucleotide in length) and its expression is downregulated in various cancers, including colorectal, liver, gastric, breast, lung, esophageal, and pancreatic cancers [121]. Recently, Paczkowska et al*.* reported that mir-148a is inactivated due to methylation in classical Hodgkin lymphoma, whereas its over expression reduces tumor cell proliferation [122]. Also, it has been shown that miR-148a inhibits tumor cell invasion, migration, and metastasis [123]. Zhang et al*.* showed that miR-148a expression is significantly downregulated in HCC tissue and cell lines, while its upregulation notably inhibits HCC cell invasion. They found that miR-148a negatively regulates S1PR_1_ in HCC cells [124].

### miR-542-3p

MiR-542-3p, as a tumor suppressor miRNA, is located on chromosome Xq26.3 which is downregulated in various types of cancers, including astrocytoma, esophageal squamous cell carcinoma, and bladder cancer [125]. For instance, Yuan et al*.* indicated that downregulation of miR-542-3p is markedly associated with lymph node metastasis and advanced tumor stage in patients with CRC. Moreover, the induced upregulation of miR-542-3p could suppress tumor cell proliferation, migration, and invasion as well as induce apoptosis both in vitro and in vivo [126]. It has been shown that there is a negative correlation between the expression levels of miR-542-3p and S1PR_1_ in breast cancer tissue and cell line. Breast cancer cells significantly reduce the expression of miR-542-3p, while the expression of S1PR_1_ is upregulated, depicting a yin yang relationship. It was demonstrated that transfection of miR-542-3p into MCF-7 cells could inhibit tumor cell proliferation, colony formation, migration, and invasion. MiR-542-3p directly binds to 3′-UTR of S1PR_1_ mRNA and suppresses its tumorigenesis activity [127].

### miR-506

MiR-506, a component of the X chromosome-linked cluster in the primate, is involved in various physiological and pathological conditions, including cancer [128, 129]. Most studies demonstrated miR-506 as a tumor suppressor miRNA in cancer such as CRC [130], lung [131], and ovarian [132] cancers. For example, it has been shown that miR-506 is markedly reduced in nasopharyngeal carcinoma tissues and cell lines. In contrast, its ectopic upregulation significantly suppresses cell proliferation, colony formation, and invasion by targeting Forkhead box Q1 (FOXQ1), which is involved in tumor cell proliferation and metastasis [133]. Lu et al*.* found that transient transfection of miR-506 in hepatoma cells restrained SphK1 expression at both mRNA and protein levels, whereas treatment with miR-506 inhibitor leads to opposite results, suggesting that miR-506 directly targets SphK1. They also indicated that inhibition of SphK1 via miR-506 suppresses tumor angiogenesis [134]. In another study, Li et al*.* indicated that downregulation of miR-506 is notably associated with pathological tumor status, clinical stage, distant metastasis, and decreased survival in patients with pancreatic cancer. Furthermore, miR-506 ectopic overexpression inhibits cell proliferation and induces cell cycle arrest, apoptosis, and chemosensitivity. They identified that tumor-suppressive effects of miR-506 are associated with targeting SphK1 and inhibition of the SphK1/Akt/NF-κB signaling pathway. They also studied the mechanism responsible for miR-506 downregulation in cancer tissues and identified that hypermethylation of one of five CpG sites in the miR-506 promoter region silences its expression in pancreatic cancer [135].

### miR‐101

MiR-101 is generated from two precursors: miR-101–1 and miR-101–2, which are located on chromosomes 1p31 and 9p24, respectively. As a tumor suppressor miRNA, miR-101 is downregulated in various cancers, including HCC, osteosarcoma, oral squamous cell carcinoma, gastric, bladder, and cervical cancer [136]. For example, miR-101 can inhibit tumor cell proliferation, growth, migration, and metastasis and enhance apoptosis and the inhibitory effect of chemotherapy agents on cancerous cells [137–139]. Chen et al*.* showed that the exogenous expression of miR-101 by CRC cell lines inhibits cell growth by targeting and inhibiting SphK1 expression, leading to ceramide production as a pro-apoptotic product. In contrast, miR-101 downregulation increases SphK1 expression and reduces ceramide levels in CRC cells. They also reported that overexpression of miR-101 increases the chemosensitivity of CRC cells both in vitro and in vivo [140]. It has been shown that S1P could suppress tumor cell migration and downregulate MMP-2 expression in chondrosarcoma cells through upregulation of tissue inhibitor of metalloproteinase‐3 (TIMP‐3), which is the target miR-101. Thus, the S1P/TIMP-3/miR-101 pathway determines the metastasis of chondrosarcoma [141].

### miR-9

There are three precursor transcripts for miR-9: miR-9-1, miR-9-2, and miR-9-3, which are located on chromosomes 1, 5, and 15, respectively [142]. MiR-9 displays opposite roles in various cancers. For instance, it has been demonstrated that the absorption of exosomal miR-9 via ECs could promote tumorigenesis and angiogenesis in glioma [143]. On the other hand, miR-9 acts as an inhibitor of cell proliferation and invasion and an inducer of apoptosis in ovarian cancer [144]. MiR-9 also enhances the sensitivity of lung cancer cells to cisplatin [145]. Recently, Yao et al*.* investigated the role of miR-9 in angiogenesis and found that miR-9 is upregulated in ECs from glioblastoma and medulloblastoma xenograft. They showed that miR-9 overexpression is negatively associated with the expression of S1PR_1_ and S1PR_3_. Although miR-9 meaningfully suppresses both S1PR_1_ and S1PR_3_ at mRNA and protein levels, only S1PR_1_ was directly targeted via miR-9. Furthermore, S1PR_1_ upregulation inhibited the miR-9-induced angiogenesis [146].

### miR-124

Three genes have been identified for human miR-124, including miR-124a-1, miR-124a-2, and miR-124a-3, located on the chromosomes 8p23.1, 8q12.3, and 20q13.33, respectively [147]. There is evidence indicating miR-124 dysregulates in various cancers and acts as a tumor suppressor miRNA. For example, Xie et al*.* found that miR-124 expression is downregulated in CRC patients, whereas its upregulation in CRC cells could intensify the cytotoxicity effects of chemotherapy agents [148]. In gastric cancer, it downregulates SPHK1 expression via direct targeting of its 3′-UTR [149]. MiR-124 also suppresses the migration and invasion of HCC cells by inhibiting integrin αV [150]. Integrins are essential transducers of bidirectional cellular signaling and regulators of cell adhesion, migration, invasion, and tissue remodeling [151, 152]. It has been demonstrated that miR-124 exerts its anti-tumor effects via targeting SphK1 in osteosarcoma, melanoma, head, and neck squamous cell carcinoma, and ovarian cancer [153–156]. The inhibition of SphK1 by miR-124 leads to ceramide accumulation, an increase in the levels of pro-apoptotic PARP, BAX, and BAD proteins, and a decrease in the levels of anti-apoptotic Bcl-xL and Bcl-2 proteins [155]. In another study, Zhang et al*.* indicated that the exosomal transfer of miR-124 hinders normal fibroblasts' transition to cancer-associated ones and cell mobility via inhibiting SphK1 in ovarian cancer [156]. So, miR-124 upregulation is a promising candidate in controlling tumor growth and metastasis.

### miR-125-b

There are two precursor transcripts for miR-125-b, including miR-125b-1 and miR-125b-2, located on the chromosomes 11q24 and 21q21. It has been shown that miR-125-b plays a dual role in tumorigenesis [157]. For example, miR-125-b is remarkably upregulated in retinoblastoma and leukemia, leading to tumor growth, drug resistance, and apoptosis suppression [158, 159]. On the other hand, the miR-125-b expression is significantly reduced in breast, ovarian, and thyroid cancer [160–162]. Zhao et al*.* investigated the expression levels and potential role of miR-125-b in the pathogenesis of bladder cancer. They indicated that patients with bladder cancer downregulate miR-125-b expression, whereas the external overexpression of miR-125-b could inhibit cellular growth, migration, and cell cycle arrest via directly targeting the 3′-UTR of SphK1 [163]. In another study, Zhang et al*.* found that patients with non-small cell lung cancer (NSCLC) and NSCLC cell lines significantly reduce the expression levels of miR-125b-1-3p. Furthermore, overexpression of miR-125b-1-3p suppresses cell proliferation, migration, and invasion and induces apoptosis of NSCLC cells by binding to the 3′-UTR of S1PR_1_ [164].

### miR-128

Two genes encode miR-128: miR-128–1 located on chromosome 2q21.3 and miR-128–2 located on chromosome 3p22.3 [165]. It has been demonstrated that miR-128 aberrantly expresses in many kinds of malignancies. Hu et al*.* reported that miR-128 expression was notably reduced in NSCLC tissues and cells, and it is correlated with cancer cell differentiation, lymph node metastasis, and pathological stage. In contrast, ectopic upregulation of miR-128 inhibited cell proliferation, colony formation, migration, invasion, and angiogenesis and induced cell cycle arrest and apoptosis [166]. The expression levels of miR-128 are remarkably downregulated in papillary thyroid cancer and follicular thyroid carcinoma tissues and cell lines, whereas its ectopic upregulation hampers tumor cell proliferation and invasion and induces cell cycle arrest and apoptosis by targeting SphK1. On the other hand, SphK1 upregulation notably abrogated anti-tumor functions of miR-128, which promotes proliferation and metastasis and reduces apoptosis. Furthermore, miR-128 overexpression in a tumor mice model reduces tumor weight and tumor growth rate and suppresses SphK1 [167]. Thus, miR-128 may be considered as an anti-tumor agent owing to its tumor-suppressive role.

### miR-613

MiR-613 is located on chromosome 12p13.1 and there is growing evidence revealing its crucial role, generally its tumor suppressor role, in tumorigenesis [168]. It has been demonstrated that the expression levels of miR-613 are significantly reduced in various cancers, including glioma, gastric, and breast cancer [169–171]. The lower levels of miR-613 were remarkably negatively associated with the overall free survival in patients with melanoma, whereas its ectopic upregulation inhibited tumor cell proliferation, colony formation, migration, and invasion via targeting the sex-determining region Y-box 9 (SOX9), which promotes cell proliferation and invasion as an oncogene [169]. Moreover, both SphK1 and SphK2 are the direct target of miR-613. MiR-613 is downregulated in bladder and papillary thyroid cancer cell lines and tissues. In contrast, its ectopic upregulation suppresses tumor cell growth, migration, invasion, and EMT by targeting the 3′-UTR of SphK1 and SphK2 in the bladder and papillary thyroid cancers, respectively [172, 173].

### miR-515-5p

MiR-515-5p is located on chromosome 19, which acts as a tumor suppressor. For instance, Zhang et al*.* found that prostate cancer cell lines and tissues significantly reduce the expression of miR-515-5p, whereas its overexpression negatively regulates tumor cell proliferation and migratory characteristics by binding to thyroid hormone receptor interactor 13 (TRIP13), as a tumor promoter. Furthermore, the expression levels of miR-515-5p in the advanced T stage of patients with prostate cancer are remarkably lower than those in the early T stage [174]. It has been shown that miR-515-5p also mediates its tumor suppressor roles via targeting SphK1. Pinho et al*.* demonstrated that estrogen receptor α (ERα) suppresses the expression of miR-515-5p in breast cancer and its enforced expression reduces tumor cell proliferation and SphK1 activity and induces caspase-dependent apoptosis. They reported that miR-515-5p downregulation increases the proliferation of breast cancer cells and estrogen-dependent SphK1 activity [175].

### miR-330-3p

**Fig. 4:**
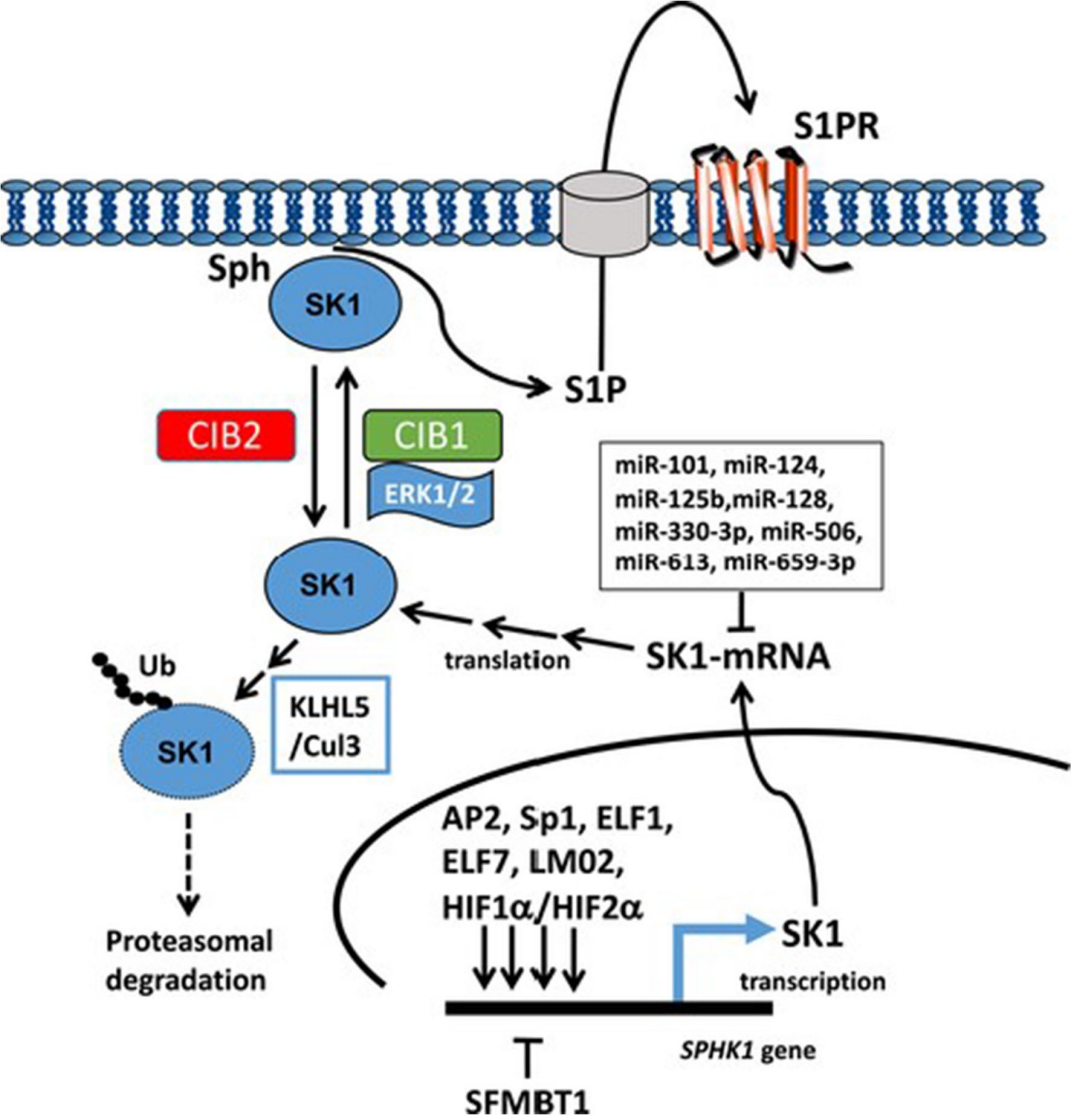
Sphingosine kinase 1 regulation in cancer. The transcriptional and post-translational processes that regulate SK1 in cancer are depicted in this diagram. AP2, Sp1, ELF1, ELF7, LM02, and HIF1a/HIF2a are involved in stimulated transcriptional regulation of SK1 gene expression, whereas SFMBT2 is involved in inhibition. SK1 is translocated to the plasma membrane after being post-translationally changed (phosphorylation) by ERK-2; translocation is favourably controlled by CIB1 and hindered by CIB2. Because SK1 is localized at the plasma membrane, it can access its substrate, resulting in the synthesis of S1P, which is subsequently released to act on S1P receptors. SK1 is regulated by KLH5-Cul3, which promotes ubiquitin-proteasomal degradation of the protein. (Adopted from [195])

MiR-330-3p, located on chromosome 19q13.32 [176], is dysregulated in various cancers. It has been demonstrated that miR-330-3p is downregulated in laryngeal squamous cell carcinoma [177] and glioma [178] but overexpressed in lung cancer [179] and esophageal squamous cell carcinoma (ESCC) [180]. For example, miR-330-3p can regulate the proliferation and migration of glioma cells as a tumor suppressor miRNA [178]. On the other hand, upregulation of miR-330-3p notably promotes proliferation, migration, invasion, and survival of ESCC cells in vitro and stimulates tumor formation in the mice model [180]. Wang et al*.* found that miR-330-3p directly targets SphK1 and S1PR_1_ in gastric cancer cells. They reported that SphK1 knockdown could remarkably suppress tumor cell proliferation, block cell cycle, induce apoptosis, and inhibit their invasiveness. Furthermore, miR-330-3p upregulation inhibits SphK1 and S1PR_1_ expression levels like FTY720 (an SphK1 inhibitor) and VPC23019 (an S1PR_1_ antagonist) [181]. There are numerous examples of SK1 (SphK1) overexpression at the mRNA and protein levels, which is frequently associated with poor prognosis in cancer patients, including shorter survival and earlier disease recurrence [5]. Triple-negative breast cancer [67], Melanoma [182], non-small cell lung cancer [183], colorectal cancer [70] and papillary thyroid carcinoma [184] are some recent examples. This is in line with SK1's propensity to enhance cell survival, proliferation, and neoplastic transformation, and it backs up SK1(SphK1) inhibitors' therapeutic potential. The molecular processes that regulate SK1 (SphK1) is abridged in Fig. 4.

### miR-144-3p

MiR-144-3p, encoded by chromosome 17q11.2 [185], plays a controversial role in various cancers. It acts as an oncogene in papillary thyroid carcinoma [186] and renal carcinoma [187], whereas it also can act as a tumor suppressor in laryngeal squamous cell carcinoma [188] and gastric cancer [189]. For instance, miR-144-3p upregulation promotes tumor cell survival, growth, and metastasis in papillary thyroid carcinoma, while it can suppress proliferation, EMT, and invasiveness of gastric cancer cells [189]. Using bioinformatics predictions and digital gene expression (DGE) analysis, Liang et al*.* found that miR-144-3p mediates the pro-invasive role of SphK1 in papillary thyroid cancer cells via targeting the fibronectin 1 (FN1) protein. They also showed that miR- 144-3p expression levels are decreased by SphK1 overexpression [190].

### miR-133b

MiR-133b, encoded by chromosome 6p12.2, is abnormally expressed in different types of cancers and plays a dual role in tumorigenesis [191]. For example, it has been shown that miR-133b is downregulated in tissues and cell lines of breast cancer, whereas its exogenous upregulation could inhibit tumorigenesis, colony formation, and metastasis characteristics of breast cancer [192]. On the other hand, miR-133b can act as an oncogene in cervical cancer, which promotes tumorigenesis and metastasis [193]. Cheng et al*.* investigated the expression, roles, and underlying mechanisms of miR-133b in nasopharyngeal carcinoma. They demonstrated that nasopharyngeal carcinoma tissues significantly reduced the expression levels of miR-133b compared with adjacent normal tissues. The exogenous miR-133b upregulation could significantly inhibit nasopharyngeal carcinoma cell proliferation via targeting S1PR_1_ and regulating STAT3 signaling. Since S1PR_1_ is crucial for STAT3 activation in cancer cells, they found that overexpression of miR-133b markedly reduced the expression levels of STAT3 in the phosphorylated form [194].

### Other miRNAs

In addition to the mentioned miRNAs, other ones also regulate SphKs, S1P, and S1PRs in several types of cancer. For instance, miR-659-3p negatively regulates SphK1 in chronic myeloid leukemia and CRC, leading to inhibition of cell proliferation, induction of apoptosis, and regulation of resistance to chemotherapy [195, 196]. MiR-659-3p expression was notably reduced in cisplatin-resistant CRC clinical samples and cell lines, whereas the expression levels of SphK1 were increased in cisplatin-resistant CRC samples. Additionally, knockdown of SphK1 could resensitize parental CRC cell lines to cisplatin [196]. Another miRNA that regulates response to therapy by targeting the S1P pathway is miR-95. Huang et al*.* found that miR-95 mediates resistance to radiotherapy by targeting S1P phosphatase 1 (SGPP1), while FTY720 treatment of tumor cells sensitized them to radiotherapy [197]. Zhang et al*.* showed that miR-338-3p expression levels are downregulated in NSCLC tissues. In contrast, miR-338-3p overexpression notably induced apoptosis and inhibited the proliferation of NSCLC cells via directly inhibiting SphK2 [198]. In another study, Bao et al*.* indicated that CRC tissues and cell lines significantly decreased miR-27a levels, which was associated with clinical pathological stages and distant metastasis, whereas its overexpression could attenuate CRC cell proliferation and migration and promote apoptosis by targeting SGPP1 and Smad2 [199]. In gastric cancer tissues and cell lines, overexpression of miR-31 remarkably reduced SGPP2 and Smad4 levels, leading to inhibition of tumor cell proliferation and migration and promotion of apoptosis [200]. We also retrieved oncogenic miRNA interactions with S1P, S1PRs, and SphKs via miRTex database (URL: https://research.bioinformatics.udel.edu/miRTex/) shown in the Table 2.

**Table 2:** miRNA interactions with S1P, S1PRs, and SphKs with a role in cancer pathogenesis

Serial no	miRNA	Regulation	Cancer type/function	Interacting/target gene	References
1	miR-506	Downregulates	Liver cancer angiogenesis	SPHK1/S1P	[134]
2	miR-17	Downregulates	Migration of thyroid cancer cells	S1P	[201]
3	miR-95	Upregulates	Tumor growth and radiation resistance in PC3 prostate and breast cancer cells	S1P and SGPP1	[197]
4	MicroRNA-363	Downregulates	Proliferation of hepatocellular carcinoma cells	S1PR1	[202]
5	microRNA-148a	Downregulates	Hepatocellular carcinoma cell invasion	S1PR1	[124]
6	miR302-367	Downregulates	Tumor growth and angiogenesis	S1PR1	[203]
7	miR-92a	Downregulates	Tumor growth and angiogenesis	S1PR1	[204]
8	MiR-101	Downregulates	Colorectal cancer cells expression	SphK1	[140]
9	MiR-124	Downregulates	Ovarian cancer cell invasion/migration	SphK1	[205]
10	miR-125b	Downregulates	Bladder cancer cell proliferation and migration	SphK1	[163]
11	miR-659-3p	Downregulates	Colorectal cancer cells	SphK1	[196]
12	miR-107	Upregulates	Tumor angiogenesis in liver cancer	SphK1	[206]
13	miR-613	Downregulates	Papillary thyroid carcinoma	SphK2	[173]

## Conclusions

In the last decades, significant effort has been expended to clarify the function of S1P signaling and its modulators, including SphKs and S1P receptors, in the development and treatment of human malignancies. The interaction between SphKs, S1P, and S1P receptors with miRNAs and their roles in tumorigenesis has attracted increasing interest in finding out this regulatory network in different aspects of cancer. The researchers recognized that targeting SphKs, S1P, and S1P receptors with different miRNAs can inhibit tumor cell proliferation, survival, migration, and metastasis. Today, SphK2 inhibitor ABC294640 has undergone clinical trials to treat pancreatic cancer and multiple myeloma (NCT01488513 and NCT02757326). Furthermore, the loss of S1PR_1_ in T-cell dysfunction for anti-tumor activities is under investigation (NCT04657146). Uncovering the crosstalk between miRNAs and the S1P pathway will provide promising outcomes in clinical trials. On the other hand, emerging novel isothermal amplification techniques for quantifying miRNAs, including duplex-specific nuclease signal amplification and rolling circle amplification, may overcome current methods' limitations by minimizing equipment demands, reducing cost and time, and providing specific and sensitive miRNA detection. The ease and speed of these techniques will help to identify the targets of miRNAs in the S1P pathway and, ultimately, proper preventive and therapeutic interventions. Therefore, fully understanding the interaction between SphKs, S1P, and S1P receptors with miRNAs in human malignancies will provide a new approach in cancer therapy based on miRNAs.

## Data Availability

Not applicable.

## Abbreviations

S1P: Sphingosine-1-phosphate
SphKs: Sphingosine kinases
S1PRs: S1P receptors
EMT: Epithelial-mesenchymal transition
miRNAs: MicroRNAs
3′-UTR: 3′-Untranslated regions
ABC: ATP-binding cassette
SPNS2: Spinster homolog 2
CRC: Colorectal cancer
HCC: Hepatocellular carcinoma
NSCLC: Non-small cell lung cancer
EC: Endothelial cell
